# SUBJECTIVE COGNITIVE DECLINE AS A PREDICTOR OF INCIDENT HOMEBOUND STATUS AMONG OLDER ADULTS

**DOI:** 10.1093/geroni/igac059.096

**Published:** 2022-12-20

**Authors:** Julia Burgdorf, Jennifer Reckrey

**Affiliations:** Center for Home Care Policy & Research, VNS Health, New York, New York, United States; Icahn School of Medicine, The Mount Sinai Hospital, New York, New York, United States

## Abstract

In the US, over 2 million older adults are homebound, meaning they never or rarely leave their home. Being homebound increases risk for negative outcomes, including depression and mortality. Dementia diagnosis is a risk factor for becoming homebound, but over half of those with dementia do not receive a formal diagnosis. Subjective cognitive decline (SCD)—self-reported increase in memory loss over a given timeframe—is an early indicator of dementia that can be easily determined during routine medical encounters. SCD screening could be a valuable tool for identifying older adults at risk of becoming homebound—a critical first step towards supporting these individuals and their caregivers—but its predictive value is not yet known. We examined a nationally representative sample of 4,914 (weighted n=34,381,060) community-living older adults who responded to the 2016 and 2017 National Health and Aging Trends Study (NHATS). We modelled incident homebound status as a function of SCD using weighted, multivariable logistic regression while adjusting for older adult sociodemographic characteristics, health status, and caregiving support. In adjusted models, those who reported SCD experienced 43% greater odds of becoming newly homebound from 2016-2017 (aOR: 1.43; p=0.03). This relationship persisted among individuals without a formal dementia diagnosis (n=4,735); among these individuals, those who reported SCD experienced 50% greater odds of becoming newly homebound (aOR: 1.50; p=0.02). Findings suggest the potential value of SCD screening as an efficient method of identifying older adults who may benefit from resources that reduce the risk and/or mitigate the negative effects of becoming homebound.

